# Generative Artificial Intelligence Tools in Medical Research (GAMER): Protocol for a Scoping Review and Development of Reporting Guidelines

**DOI:** 10.2196/64640

**Published:** 2025-08-14

**Authors:** Xufei Luo, Yih Chung Tham, Mohammad Daher, Zhaoxiang Bian, Yaolong Chen, Janne Estill, GAMER Working Group

**Affiliations:** 1 Evidence-Based Medicine Center School of Basic Medical Sciences Lanzhou University Lanzhou China; 2 Research Unit of Evidence-Based Evaluation and Guidelines, Chinese Academy of Medical Sciences (2021RU017) School of Basic Medical Sciences Lanzhou University Lanzhou China; 3 Institute of Health Data Science Lanzhou University Lanzhou China; 4 World Health Organization Collaboration Center for Guideline Implementation and Knowledge Translation Lanzhou China; 5 Key Laboratory of Evidence Based Medicine of Gansu Province Lanzhou University Lanzhou China; 6 Centre for Innovation and Precision Eye Health National University of Singapore Singapore Singapore; 7 Singapore National Eye Centre Singapore Eye Research Institute Singapore Singapore; 8 Ophthalmology and Visual Science Academic Clinical Program Duke-National University of Singapore Medical School Singapore Singapore; 9 Department of Ophthalmology National University of Singapore Singapore Singapore; 10 Orthopedics Department Hotel Dieu de France Beirut Lebanon; 11 Vincent VC Woo Chinese Medicine Clinical Research Institute School of Chinese Medicine Hong Kong Baptist University Hong Kong China; 12 Chinese Enhancing the Quality and Transparency of Health Research (EQUATOR) Centre Hong Kong China; 13 Institute of Global Health University of Geneva Geneva Switzerland; 14 See Acknowledgments

**Keywords:** generative AI, chatbots, reporting guidelines, transparency, Delphi method, large language models, ChatGPT

## Abstract

**Background:**

The integration of artificial intelligence (AI) has revolutionized medical research, offering innovative solutions for data collection, patient engagement, and information dissemination. Powerful generative AI (GenAI) tools and other similar chatbots have emerged, facilitating user interactions with virtual conversational agents. However, the increasing use of GenAI tools in medical research presents challenges, including ethical concerns, data privacy issues, and the potential for generating false content. These issues necessitate standardization of reporting to ensure transparency and scientific rigor.

**Objective:**

The development of the Generative Artificial Intelligence Tools in Medical Research (GAMER) reporting guidelines aims to establish comprehensive, standardized guidelines for reporting the use of GenAI tools in medical research.

**Methods:**

The GAMER guidelines are being developed following the methodology recommended by the Enhancing the Quality and Transparency of Health Research (EQUATOR) Network, involving a scoping review and expert Delphi consensus. The scoping review searched PubMed, Web of Science, Embase, CINAHL, PsycINFO, and Google Scholar (for the first 200 results) using keywords like “generative AI” and “medical research” to identify reporting elements in GenAI-related studies. The Delphi process involves 30-50 experts with ≥3 years of experience in AI applications or medical research, selected based on publication records and expertise across disciplines (eg, clinicians and data scientists) and regions (eg, Asia and Europe). A 7-point-scale survey will establish consensus on checklist items. The testing phase invites authors to apply the GAMER checklist to GenAI-related manuscripts and provide feedback via a questionnaire, while experts assess reliability (κ statistic) and usability (time taken, 7-point Likert scale). The study has been approved by the Ethics Committee of the Institute of Health Data Science at Lanzhou University (HDS-202406-01).

**Results:**

The GAMER project was launched in July 2023 by the Evidence-Based Medicine Center of Lanzhou University and the WHO Collaborating Centre for Guideline Implementation and Knowledge Translation, and it concluded in July 2024. The scoping review was completed in November 2023. The Delphi process was conducted from October 2023 to April 2024. The testing phase began in March 2025 and is ongoing. The expected outcome of the GAMER project is a reporting checklist accompanied by relevant terminology, examples, and explanations to guide stakeholders in better reporting the use of GenAI tools.

**Conclusions:**

GAMER aims to guide researchers, reviewers, and editors in the transparent and scientific application of GenAI tools in medical research. By providing a standardized reporting checklist, GAMER seeks to enhance the clarity, completeness, and integrity of research involving GenAI tools, thereby promoting collaboration, comparability, and cumulative knowledge generation in AI-driven health care technologies.

**International Registered Report Identifier (IRRID):**

DERR1-10.2196/64640

## Introduction

Generative artificial intelligence (GenAI)–based tools and chatbots have been widely applied in medicine, facilitating interactions between users and AI through virtual conversational agents and thereby presenting new opportunities for enhancing health care practice and research methodology [1,2]. The increasing use of GenAI tools linked to chatbots in medical research brings numerous opportunities and supports innovations, but it also poses many challenges and creates issues. For example, the use of large language models may raise ethical concerns and data privacy issues [3,4]. Additionally, the generation of false content using large language models can constitute academic fraud [5]. These problems have detrimental effects on the academic community. Therefore, transparent and standardized reporting of the use of GenAI tools is extremely important. Although many journals and academic institutions have their own guidelines or suggestions on how to use GenAI tools [6-10], there are currently no universally recognized reporting guidelines on how to transparently and scientifically report the use of GenAI tools in medical research.

The Generative Artificial Intelligence Tools in Medical Research (GAMER) project aims to establish comprehensive and standardized guidelines for reporting essential elements of studies involving GenAI-based tools. The GAMER guidelines will delineate key aspects, such as whether GenAI tools were used in the study and in the writing of the paper, which tool and version were used, which sections of the paper it was applied to, the specific content areas it was used for, whether it was used for language editing or content generation, and whether any manual testing was performed. By providing recommendations for what information should be reported for each section, GAMER seeks to create reporting guidelines that streamline the reporting process, allowing researchers, practitioners, and policymakers to critically assess the quality and validity of studies involving GenAI tools in medical research [3].

The development of GAMER draws inspiration from established reporting guidelines in the field of medical research, such as the CONSORT (Consolidated Standards of Reporting Trials) statement for clinical trials [11] and the STROBE (Strengthening the Reporting of Observational Studies in Epidemiology) statement for observational studies [12]. Adhering to GAMER will not only enhance the clarity and completeness of reporting in GenAI-related research but will also foster collaboration, comparability, and cumulative knowledge generation in the rapidly evolving field of AI-driven health care technologies. This article describes the methods and processes involved in the development of GAMER, providing a guarantee for the smooth completion and promotion of GAMER in the future.

## Methods

We will follow the methodology recommended by the EQUATOR (Enhancing the Quality and Transparency Of Health Research) Network [13] to develop the GAMER reporting guidelines, using methods such as scoping reviews and expert Delphi consensus to formulate the GAMER reporting checklist. We have registered this protocol with the EQUATOR Network [14].

### Project Administration

The GAMER reporting guidelines were initiated by the Evidence-Based Medicine Center of Lanzhou University, the Chinese EQUATOR Center, and the WHO Collaborating Centre for Guideline Implementation and Knowledge Translation. Authors YC and JE are the co–project initiators, and author XL serves as the lead investigator and the main contact for communication.

### Funding Sources

The project is funded by research project funds from the Research Unit of Evidence-Based Evaluation and Guidelines (2021RU017), Chinese Academy of Medical Sciences; and the School of Basic Medical Sciences, Lanzhou University, with no involvement of pharmaceutical companies or other third-party entities. The funding is used solely for relevant services, materials, travel, and publication expenses during the development process of GAMER.

### Scope and Target Audience

The scope of GAMER is to assist stakeholders such as researchers, clinicians, editors, authors, peer reviewers, and other relevant parties in the smooth and barrier-free use and evaluation of GenAI tools and similar chatbots for the purpose of composing medical research.

### Development Process

**Figure 1:**
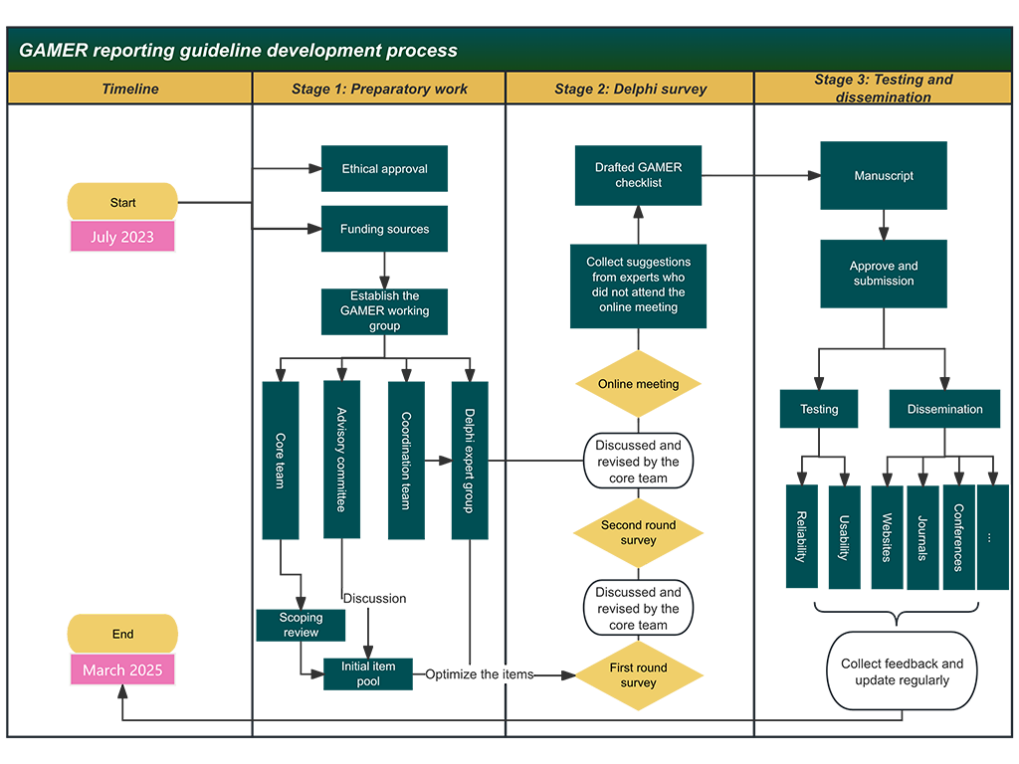
The 3 stages of development of the GAMER (Generative Artificial Intelligence Tools in Medical Research) reporting guideline.

The development of the GAMER reporting checklist has 3 stages (Figure 1). The first stage is the preparatory phase, involving the formation of an expert group, collection of initial items, and discussion of these items. The second stage primarily includes 1 to 2 rounds of Delphi survey and online, face-to-face discussions, as well as multiple rounds of optimizing items. The third stage involves the publication of the checklist, as well as testing, dissemination, and promotion of the GAMER reporting guidelines.

#### Stage 1: Preparatory Work

##### Establishment of the Expert Panel

We plan to establish 4 expert groups. The first is the advisory committee, consisting of 3 to 5 authoritative experts familiar with GenAI tools and experienced in the formulation of reporting guidelines. Their main responsibility is to provide advice on issues arising during the development of reporting guidelines. The second is the core team, primarily composed of members from the Evidence-Based Medicine Center at Lanzhou University. This group is responsible for conducting scoping reviews, generating the initial pool of items, revising the items, and drafting the final document. This group is the leading team for GAMER development. The third is the Delphi expert group, comprising experts with experience in a broad range of relevant disciplines from around the world. The inclusion criteria for invitation to this group are (1) participation in the publication of GenAI-related papers (obtained by searching PubMed for corresponding authors of relevant papers), supplemented by snowball sampling (experts recommending peers); (2) expertise (defined as ≥3 years of experience in AI applications or medical research) in medical-related disciplines (eg, bioinformatics, epidemiology, clinical science, data science, ethics); (3) willingness to participate in and support the development of GAMER; and (4) having no severe academic or financial conflicts of interests. We aim for 30 to 50 participants, targeting representation across disciplines (eg, including clinicians, data scientists, and ethicists) and regions (Asia, Europe, and the Americas). The Delphi expert group’s responsibilities include proposing modifications to the GAMER checklist, voting, and determining the final modifications to the full document. The fourth expert group is the coordination team, which will include 3 to 5 members. This group is responsible for coordinating the development work, communicating with experts, organizing the meetings, sending emails, and organizing materials.

##### Scoping Review

We conducted a scoping review to identify key reporting elements in studies involving GenAI tools in medical research. The primary research question was as follows: What are the essential elements that should be reported when using GenAI tools in medical research? We searched PubMed, Web of Science, Embase, CINAHL, PsycINFO, and the first 200 results of Google Scholar using keywords such as “generative AI,” “chatbots,” “ChatGPT,” “large language model,” and “reporting guidelines.” We included existing AI-related reporting guidelines that address the use of GenAI tools in medical research. Studies were eligible if they focused on the application of GenAI tools in a medical context and provided reporting recommendations or considerations. Two reviewers independently screened titles and abstracts, followed by full-text review for eligibility. Data were extracted with a focus on reporting elements and synthesized thematically to identify common themes and recommendations. The review identified key themes, such as the need to specify the GenAI tool and version used, the purpose of its application, and verification processes, which formed the initial pool of items for the Delphi survey and informed subsequent stages of GAMER development. The scoping review was submitted for publication in November 2023 and as of June 2025 is currently under review. The PRISMA-ScR (Preferred Reporting Items for Systematic Reviews and Meta-Analyses Extension for Scoping Reviews) checklist is provided in Multimedia Appendix 1.

#### Stage 2: Delphi Survey

We will follow the Accurate Consensus Reporting Document (ACCORD) guidelines for the Delphi survey [15]. Once the preliminary items are completed, a survey will be disseminated through SurveyMonkey to reach consensus using a modified Delphi method [15,16]. We anticipate conducting 1 to 3 rounds, but there is no limit, and the decision on the number of rounds will depend on whether consensus is reached in the first round. Each initial item will be assigned a survey option ranging from 1 to 7 points, with 1 indicating strong disagreement, 4 indicating neutrality, and 7 indicating strong agreement. All statistics will be based on median scores. If the median score is between 1 and 3, the item will be excluded. If the score is 4 or 5 (or higher with substantial comments on the content), the item will be discussed and entered into the next round of the Delphi survey or the consensus meeting. If the score is 6 or 7 without any substantial comments, the item will be included in the final checklist. Items unresolved after 2 rounds will be discussed in the consensus meeting for final resolution. Participants can propose new items via free-text comments. New items receiving ≥50% support from the core team will enter subsequent rounds. Abstentions are allowed. Forced responses will not be used to avoid bias. Online meetings will be held after the Delphi survey to collect the experts’ opinions and optimize and formulate the final checklist. All opinions and suggestions will be documented, and responses to each expert’s suggestions will be provided through email. Qualitative data will undergo thematic analysis. Key themes will inform item revisions, with examples provided in supplementary materials. The initial Delphi survey included 10 items derived from the scoping review, covering aspects such as the specific GenAI tool used, the version of the tool, the sections of the manuscript where GenAI was applied, the purpose of GenAI use (eg, language editing or content generation), and whether manual verification was performed.

After the Delphi survey and virtual consensus meeting, we will refine and optimize the final items included. Once approved by the experts, we will prepare the manuscript for submission.

#### Stage 3: Testing and Dissemination

After the GAMER checklist is finalized, we will conduct a real-life implementation testing phase. Authors of medical research papers involving GenAI tools will be invited to apply the checklist during manuscript preparation and provide feedback via a structured questionnaire developed to assess clarity, completeness, and ease of use. Concurrently, experts from various medical disciplines will evaluate reliability and usability. For reliability, multiple experts will independently apply the checklist to a sample of GenAI-related papers, with interrater agreement measured using the κ statistic. For usability, experts will record completion time and rate item difficulty on a 7-point Likert scale. Feedback from both authors and experts will be analyzed thematically and quantitatively to identify areas for improvement, guiding revisions to the checklist’s items, explanations, or examples for enhanced clarity and applicability. Public input will also be sought to ensure broader relevance. Explanatory documents and examples will accompany the final checklist.

Furthermore, we plan to invite the consensus expert group members to translate GAMER into their local languages and promote the dissemination of the checklist. Additionally, we will actively reach out to journals, inviting them to feature GAMER in authors’ instructions. Finally, we aim to establish a GAMER website to regularly update users on the latest developments and research findings related to the GAMER reporting guidelines.

### Timeline

The process and anticipated timeline for developing the GAMER reporting guidelines are shown in Table 1.

**Table 1:** Timeline for the development of the GAMER (Generative Artificial Intelligence Tools in Medical Research) reporting guidelines.

Tasks	2023 (month)							2024 (month)													2025 (month)		
	7	8	9	10	11	12	1		2	3	4	5	6	7	8	9	10	11	12	1		2	3
Launch the project	✓																						
Registration	✓																						
Recruit members		✓	✓																				
Scoping review		✓	✓	✓	✓																		
Draft the preliminary items				✓	✓																		
First round of Delphi survey					✓																		
Revise, discuss, and refine the checklist						✓	✓		✓														
Second round of Delphi survey										✓	✓	✓											
Online meeting												✓											
Collect feedback													✓	✓	✓								
Draft full text																✓	✓	✓					
Refine and approve full text																			✓	✓		✓	
Submission																							✓
Test																							✓
Dissemination																							✓

### Ethical Considerations

This study was approved by the Ethics Committee of the Institute of Health Data Science, Lanzhou University (HDS-202406-01). All participants in the Delphi survey and testing phase provided informed consent, with the option to opt out at any time. Data collected from participants were anonymized to ensure privacy and confidentiality, with no identifiable information stored. No compensation was provided to participants. This manuscript includes no images or data that could identify individuals; thus, no consent for image use was required.

## Results

The expected outcome of the GAMER project is a reporting checklist, accompanied by relevant terminology, examples, and explanations, to guide stakeholders in better reporting the use of GenAI tools. The GAMER project commenced on July 1, 2023, and concluded in July 2024. The scoping review was completed in November 2023 and submitted for publication. The Delphi process, involving 44 experts from 23 countries, was conducted from October 2023 to April 2024, yielding a 9-item checklist (Multimedia Appendix 2). The testing phase began in March 2025 and is ongoing, with authors and experts providing feedback. The final GAMER guideline is anticipated to be published in the second half of 2025, with dissemination to follow via journals and a dedicated website.

## Discussion

We will follow the EQUATOR Network’s recommended methodology to develop the GAMER reporting guidelines [13], inviting experts from various disciplines and with a broad geographical distribution to ensure the panel’s representativeness. We hope that GAMER can guide and assist those who use GenAI tools in their research to report and apply these tools transparently and scientifically, thereby ensuring the integrity of the research. Additionally, we hope that GAMER will guide reviewers and editors in identifying and assessing manuscripts that have applied GenAI tools, ensuring that manuscripts meet journal requirements for transparent reporting.

The development of GAMER is not only a response to the current use of GAI tools in medical research but also a forward-looking initiative. As AI technologies continue to evolve, the standardization introduced by GAMER will serve as a foundation for future advancements. The checklist’s scope, targeting various stakeholders, including researchers, clinicians, editors, peer reviewers, and authors, positions GAMER as a versatile tool with far-reaching implications. GAMER will guide reporting in studies using GenAI for tasks like data synthesis, manuscript drafting, and image generation. The methodology, drawing from the EQUATOR guidelines, ensures a rigorous and evidence-based approach, further solidifying the credibility of GAMER.

The outlined plan for the dissemination and promotion of GAMER demonstrates a comprehensive strategy to ensure widespread adoption of the reporting guidelines. By involving authors of medical research papers, collecting feedback, and actively engaging with journals, the GAMER project aims to integrate the checklist seamlessly into the publication process. The creation of a dedicated website further emphasizes the commitment to transparency and continuous improvement. This multifaceted approach is essential for the successful implementation of reporting guidelines, as it addresses both the supply side (researchers and authors) and the demand side (journals and users).

However, this study also has some limitations. First, we did not conduct a pilot test before the Delphi survey, as we considered that the items had been thoroughly discussed within the core expert panel. Second, we did not include patient and public representatives in the Delphi survey due to their limited knowledge of AI and medical topics. However, we will invite patient advocacy representatives to the advisory committee and include public feedback during the testing phase.

In conclusion, the GAMER project holds promise not only to address the immediate needs of standardizing the reporting of GenAI tool use, but also to shape the future landscape of medical research. The meticulous planning, expert involvement, and strategic approach to dissemination position GAMER as a valuable resource for researchers, practitioners, and policymakers in the dynamic realm of AI-driven health care technologies.

## Appendix

Multimedia Appendix 1 PRISMA-ScR checklist.

Multimedia Appendix 2 Nine-item checklist for the GAMER (Generative Artificial Intelligence Tools in Medical Research) reporting guidelines.

## Abbreviations

ACCORD: Accurate Consensus Reporting Document
CONSORT: Consolidated Standards of Reporting Trials
EQUATOR: Enhancing the Quality and Transparency Of Health Research
GAMER: Generative Artificial intelligence Tools in Medical Research
GenAI: generative artificial intelligence
PRISMA-ScR: Preferred Reporting Items for Systematic Reviews and Meta-Analyses Extension for Scoping Reviews
STROBE: Strengthening the Reporting of Observational Studies in Epidemiology

## Data Availability

Data are not available to share due to Department of Veterans Affairs and ethical retractions; however, annotation instructions and vocabulary are available upon request..
