# Supplementary material for: Generative Artificial Intelligence Tools in Medical Research (GAMER): Protocol for a Scoping Review and Development of Reporting Guidelines
Source: JMIR Res Protoc. 2025 Aug 14;14:e64640. doi: 10.2196/64640 (PMC12395103; doi:10.2196/64640)
Supplement: Multimedia Appendix 2 [file resprot_v14i1e64640_app2.docx]

**Multimedia Appendix 2 nine-item checklist for the GAMER reporting guideline**

| **No.** | **Item** | **Reported** | **Page** |
| --- | --- | --- | --- |
| 1 | Did you use any generative AI-based tools (such as large language models or large visual models) in any section or step of this manuscript or study? | □Yes □No □N/A |  |
| 2 | Specify the generative AI-based tool(s) used, their versions and/or release dates, and the date(s)/period the tools were used. | □Yes □No □N/A |  |
| 3 | Describe whether a specific prompting technique was used to generate any content of the manuscript or to perform analyses during the study. Please also provide the unedited responses to the prompts. | □Yes □No □N/A |  |
| 4 | If a new generative AI-based tool was developed or fine-tuned based on an existing AI model, report the name and version of the original model. | □Yes □No □N/A |  |
| 5 | Describe the role of generative AI-based tools in all phases of this study where they were used (including manuscript writing). | □Yes □No □N/A |  |
| 6 | Report the specific section or paragraphs of the manuscript that generative AI-based tools contributed to. | □Yes □No □N/A |  |
| 7 | Describe how the content generated by generative AI-based tools was verified and (when necessary) modified. | □Yes □No □N/A |  |
| 8 | Describe how data privacy and confidentiality were ensured during the use of generative AI-based tools. | □Yes □No □N/A |  |
| 9 | Describe whether and how the use of generative AI-based tools may have influenced the interpretation of results, the study’s overall accuracy, or conclusions. | □Yes □No □N/A |  |

N/A: not applicable.
